# Supplementary material for: Changes in the structure and composition of the ‘Mexical’ scrubland bee community along an elevational gradient
Source: PLoS One. 2021 Jul 1;16(7):e0254072. doi: 10.1371/journal.pone.0254072 (PMC8248643; doi:10.1371/journal.pone.0254072)
Supplement: S2 Appendix — (DOCX) [file pone.0254072.s002.docx]

**S2 Appendix. Correlations among all response and explanatory variables**.

Abbreviations

Flow_ab=Flower density (flower/m^2^)

Flow_rich=Flower species richness

Bee_ab=Bee abundance

Bee_rich=Bee species richness

ALT_m=Altitude (in meters)

MAP=Mean Annual Precipitation (mm)

MAT=Mean Annual Temperature (°C)
