# Supplementary material for: Changes in the structure and composition of the ‘Mexical’ scrubland bee community along an elevational gradient
Source: PLoS One. 2021 Jul 1;16(7):e0254072. doi: 10.1371/journal.pone.0254072 (PMC8248643; doi:10.1371/journal.pone.0254072)
Supplement: S3 Appendix — (DOCX) [file pone.0254072.s003.docx]

**S3 Appendix. List of species and/or morphospecies**.

| # sp | Family | *Genus & species or morphospecies* | number of individuals |
| --- | --- | --- | --- |
| 1 | Andrenidae | *Andrena sp1* | 1 |
| 2 | Andrenidae | *Andrena sp2* | 1 |
| 3 | Megachilidae | *Anthidiellum sp1* | 1 |
| 4 | Apidae | *Anthophora sp1* | 1 |
| 5 | Apidae | *Anthophora sp2* | 3 |
| 6 | Halictidae | *Augochlorini sp1* | 14 |
| 7 | Halictidae | *Augochlorini sp2* | 2 |
| 8 | Halictidae | *Augochlorini sp3* | 2 |
| 9 | Halictidae | *Augochlorini sp4* | 3 |
| 10 | Halictidae | *Augochlorini sp5* | 1 |
| 11 | Halictidae | *Augochloropsis ignita* | 1 |
| 12 | Halictidae | *Augochloropsis metallica* | 8 |
| 13 | Apidae | *Bombus ephippiatus* | 1 |
| 14 | Andrenidae | *Calliopsis sp1* | 5 |
| 15 | Apidae | *Centris sp1* | 3 |
| 16 | Apidae | *Centris sp2* | 1 |
| 17 | Apidae | *Centris sp3* | 2 |
| 18 | Apidae | *Ceratina (Calloceratina) eximia* | 1 |
| 19 | Apidae | *Ceratina sp1* | 3 |
| 20 | Apidae | *Ceratina sp2* | 3 |
| 21 | Apidae | *Ceratina sp3* | 10 |
| 22 | Apidae | *Ceratina sp4* | 12 |
| 23 | Apidae | *Ceratina sp4b* | 16 |
| 24 | Apidae | *Ceratina sp6* | 2 |
| 25 | Apidae | *Ceratina sp7* | 1 |
| 26 | Apidae | *Eucerini sp1* | 4 |
| 27 | Apidae | *Eucerini sp2* | 1 |
| 28 | Apidae | *Eucerini sp3* | 7 |
| 29 | Apidae | *Eucerini sp4* | 1 |
| 30 | Apidae | *Eucerini sp5* | 3 |
| 31 | Apidae | *Eucerini sp6* | 20 |
| 32 | Apidae | *Exomalopsis sp1* | 1 |
| 33 | Apidae | *Exomalopsis sp2* | 1 |
| 34 | Apidae | *Exomalopsis sp3* | 1 |
| 35 | Apidae | *Exomalopsis sp4* | 1 |
| 36 | Apidae | *Habropoda sp1* | 1 |
| 37 | Halictidae | *Halictus sp1a* | 35 |
| 38 | Halictidae | *Halictus sp1b* | 32 |
| 39 | Halictidae | *Halictus sp2* | 2 |
| 40 | Halictidae | *Halictus sp3* | 2 |
| 41 | Halictidae | *Halictus sp4* | 3 |
| 42 | Colletidae | *Hylaeus sp1* | 1 |
| 43 | Halictidae | *Lasioglossum (Dialictus) sp1* | 306 |
| 44 | Halictidae | *Lasioglossum (Dialictus) sp2* | 65 |
| 45 | Halictidae | *Lasioglossum (Lasioglossum) sp1* | 283 |
| 46 | Halictidae | *Lasioglossum sp3* | 2 |
| 47 | Megachilidae | *Lithurge sp1* | 2 |
| 48 | Andrenidae | *Macrotera sp1* | 535 |
| 49 | Andrenidae | *Macrotera sp2* | 9 |
| 50 | Megachilidae | *Megachile sp1* | 2 |
| 51 | Megachilidae | *Megachile sp2* | 1 |
| 52 | Megachilidae | *Megachile sp3* | 1 |
| 53 | Megachilidae | *Osmia aliciae* | 1 |
| 54 | Andrenidae | *Protandrena sp1* | 10 |
| 55 | Megachilidae | *Protosmia sp1* | 3 |
| 56 | Andrenidae | *Pseudopanurgus sp1* | 56 |
| 57 | Andrenidae | *Pseudopanurgus sp2* | 6 |
| 58 | Andrenidae | *Pseudopanurgus sp3* | 2 |
| 59 | Halictidae | *Sphecodes sp1* | 2 |
| 60 | Halictidae | *Sphecodes sp2* | 1(*) |
| 61 | Apidae | *Xylocopa sp1* | 1(*) |
|  |  | TOTAL SOLITARY BEES | 1502 |
|  |  |  |  |
| 62 | Apidae | *Apis mellifera* | 226 |
|  |  |  |  |
|  |  | **TOTAL BEE INDIVIDUALS** | **1728** |

(*) individuals captured out of surveys
