# Supplementary material for: Changes in the structure and composition of the ‘Mexical’ scrubland bee community along an elevational gradient
Source: PLoS One. 2021 Jul 1;16(7):e0254072. doi: 10.1371/journal.pone.0254072 (PMC8248643; doi:10.1371/journal.pone.0254072)
Supplement: S4 Appendix — excluding sigletons and doubletons, and for binary data (considering elevation as a continuous variable). (DOCX) [file pone.0254072.s004.docx]

**S4 Appendix. Results of multivariate analyses (community composition vs different variables) considering elevation as a continuous variable.**

**Table A.** Community composition vs Elevation (as a continuous variable), geographical distance, climatic variables and flower variables for quantitative matrix excluding singletons.

First, we present a Mantel test result, checking the effect of geographic distance explaining community composition dissimilarities. In Table A1, we present the results of PERMANOVA (considering only Elevation as explanatory variable) and dbRDA analyses (considering Elevation + geographic distance variables (pcnm1 and pcnm6)). Then, in Table A2, climatic variables (Mean Annual Temperature and Mean Annual Precipitation) and flower variables (flower richness and flower density) (in PERMANOVA), or climatic and flower variables + geographic distance variables (pcnm1 and pcnm6) are considered as explanatory variables. In all cases community composition is response variable, and quantitative matrix and Bray-Curtis dissimilarity index is applied.

Mantel test: r=0.44, p=0.001

| **Table A1. Only Elevation (+ geographical distance) as explanatory variables** | | | | | |
| --- | --- | --- | --- | --- | --- |
| **PERMANOVA** |  |  |  |  |  |
| variable | Df | Sum of Squares | R^2^ | F | P(>F) |
| Elevation | 1 | 0.696 | 0.233 | 5.173 | **0.001** |
| Residual | 17 | 2.289 | 0.766 |  |  |
| Total | 18 | 2.985 | 1 |  |  |
| **dbRDA** (controlling for geographic distance) | | |  |  |  |
| variable | Df | Sum of Squares | R^2^ | F | P(>F) |
| pcnm1 | 1 | 0.273 | 0.091 | 2.391 | **0.036** |
| pcnm6 | 1 | 0.298 | 0,100 | 2.619 | **0.030** |
| Elevation | 1 | 0.250 | 0.084 | 2.197 | **0.047** |
| Residual | 15 | 1.711 | 0,573 |  |  |
| Total | 18 | 2.985 | 1 |  |  |
| **Table A2. Climatic and flower variables (+geographical distance) as explanatory variables** | | | | | |
| **PERMANOVA** |  |  |  |  |  |
| variable | Df | Sum of Squares | R^2^ | F | P(>F) |
| Mean Annual Temperature (ºC) | 1 | 0.4 | 0.13 | 3.77 | **0.009** |
| Flower density | 1 | 0.36 | 0.12 | 3.42 | **0.010** |
| Flower species richness | 1 | 0.13 | 0.05 | 1.27 | 0.25 |
| Mean Annual Precipitation (mm) | 1 | 0.16 | 0,05 | 1.48 | 0.17 |
| Residual | 14 | 1.49 | 0.49 |  |  |
| **dbRDA** (controlling for geographic distance) | | |  |  |  |
| variable | Df | Sum of Squares | R^2^ | F | P(>F) |
| pcnm1 | 1 | 0.13 | 0.043 | 1.34 | 0.24 |
| pcnm6 | 1 | 0.23 | 0.076 | 2.38 | **0.033** |
| Mean Annual Temperature (ºC) | 1 | 0.27 | 0.091 | 2.83 | **0.019** |
| Flower density | 1 | 0.29 | 0.098 | 3.08 | **0.016** |
| Flower species richness | 1 | 0.14 | 0.048 | 1.53 | 0.18 |
| Mean Annual Precipitation (mm) | 1 | 0.02 | 0.007 | 0.24 | 0.97 |
| Residual | 12 | 1.14 | 0.38 |  |  |

**Table B**. Community composition vs Elevation (as a continuous variable), geographical distance, climatic variables and flower variables for quantitative matrix excluding singletons and doubletons.

First, we present a Mantel test result, checking geographic distance effect explaining community composition dissimilarities. In Table B1, we present the results of PERMANOVA (considering only Elevation as explanatory variable) and dbRDA analyses (considering Elevation + geographic distance variables (pcnm1 and pcnm6)). Then, in Table B2., climatic variables (Mean Annual Temperature and Mean Annual Precipitation) and flower variables (flower richness and flower density) (in PERMANOVA), or climatic and flower variables + geographic distance variables (pcnm1 and pcnm6) are considered as explanatory variables. In all cases community composition is response variable, and quantitative matrix and Bray-Curtis dissimilarity index is applied.

Mantel test: r=0.438, p=0.001

| **Table B1. Only Elevation (+ geographical distance) as explanatory variables** | | | | | |
| --- | --- | --- | --- | --- | --- |
| **PERMANOVA** |  |  |  |  |  |
| variable | Df | Sum of Squares | R^2^ | F | P(>F) |
| Elevation | 1 | 0.688 | 0.235 | 5.244 | **0.002** |
| Residual | 17 | 2.233 | 0.764 |  |  |
| Total | 18 | 2.922 | 1 |  |  |
| **dbRDA** (controlling for geographic distance) | | |  |  |  |
| variable | Df | Sum of Squares | R^2^ | F | P(>F) |
| pcnm1 | 1 | 0.265 | 0.090 | 2.405 | **0.044** |
| pcnm6 | 1 | 0.305 | 0.104 | 2.765 | **0.028** |
| Elevation | 1 | 0.244 | 0.083 | 2.212 | **0.040** |
| Residual | 15 | 1.656 | 0.566 |  |  |
| Total | 18 | 2.922 | 1 |  |  |
| **Table B2. Climatic and flower as explanatory variables** | | | | | |
| **PERMANOVA** |  |  |  |  |  |
| variable | Df | Sum of Squares | R^2^ | F | P(>F) |
| Mean Annual Temperature (ºC) | 1 | 0.39 | 0.13 | 3.84 | **0.008** |
| Flower density | 1 | 0.37 | 0.13 | 3.57 | **0.011** |
| Flower species richness | 1 | 0.13 | 0.04 | 1.26 | 0.255 |
| Mean Annual Precipitation (mm) | 1 | 0.15 | 0.53 | 1.51 | 0.151 |
| Residual | 14 | 1.44 | 0.49 |  |  |
| **dbRDA** (controlling for geographic distance) | | |  |  |  |
| variable | Df | Sum of Squares | R^2^ | F | P(>F) |
| pcnm1 | 1 | 0.12 | 0.041 | 1.31 | 0.269 |
| pcnm6 | 1 | 0.23 | 0.078 | 2.51 | **0.034** |
| Mean Annual Temperature (ºC) | 1 | 0.26 | 0.091 | 2.91 | **0.022** |
| Flower density | 1 | 0.29 | 0.1 | 3.21 | **0.014** |
| Flower species richness | 1 | 0.14 | 0.048 | 1.54 | 0.162 |
| Mean Annual Precipitation (mm) | 1 | 0.01 | 0.006 | 0.17 | 0.986 |
| Residual | 12 | 1.10 | 0.375 |  |  |

**Table C.** Community composition vs Elevation (as a continuous variable), geographical distance, climatic variables and flower variables, for qualitative (binary) matrix.

First, we present a Mantel test result, checking geographic distance effect explaining community composition dissimilarities. In Table C1., we present the results of PERMANOVA (considering only Elevation as explanatory variable) and dbRDA analyses (considering Elevation + geographic distance variables (pcnm1 and pcnm6)). Then, in Table C2., climatic variables (Mean Annual Temperature and Mean Annual Precipitation) and flower variables (flower richness and flower density) (in PERMANOVA), or climatic and flower variables + geographic distance variables (pcnm1 and pcnm6) are considered as explanatory variables. In all cases community composition is response variable, and quantitative matrix and Bray-Curtis dissimilarity index is applied.

Mantel test: r=0.318, p=0.003

| **Table C1. Only Elevation (+ geographical distance) as explanatory variables** | | | | | |
| --- | --- | --- | --- | --- | --- |
| **PERMANOVA** |  |  |  |  |  |
| variable | Df | Sum of Squares | R^2^ | F | P(>F) |
| Elevation | 1 | 0.654 | 0.153 | 3.087 | **0.001** |
| Residual | 17 | 3.601 | 0.846 |  |  |
| Total | 18 | 4.255 | 1 |  |  |
| **dbRDA** (controlling for geographic distance) | | |  |  |  |
| variable | Df | Sum of Squares | R^2^ | F | P(>F) |
| pcnm1 | 1 | 0.264 | 0.062 | 1.304 | 0.171 |
| pcnm5 | 1 | 0.298 | 0.070 | 1.468 | 0.079 |
| Elevation | 1 | 0.338 | 0.079 | 1.668 | **0.034** |
| Residual | 15 | 3.044 | 0.715 |  |  |
| Total | 18 | 4.255 | 1 |  |  |
| **Table C2. Climatic and flower as explanatory variables** | | | | | |
| **PERMANOVA** |  |  |  |  |  |
| variable | Df | Sum of Squares | R^2^ | F | P(>F) |
| Mean Annual Temperature (ºC) | 1 | 0.45 | 0.11 | 2.21 | **0.007** |
| Flower density | 1 | 0.2 | 0.047 | 0.98 | 0.46 |
| Flower species richness | 1 | 0.24 | 0.056 | 1.18 | 0.25 |
| Mean Annual Precipitation (mm) | 1 | 0.24 | 0.056 | 1.17 | 0.25 |
| Residual | 14 | 2.84 | 0.66 |  |  |
| **dbRDA** (controlling for geographic distance) | | |  |  |  |
| variable | Df | Sum of Squares | R^2^ | F | P(>F) |
| pcnm1 | 1 | 0.19 | 0.044 | 0.96 | 0.495 |
| pcnm5 | 1 | 0.33 | 0.077 | 1.69 | **0.036** |
| Mean Annual Temperature (ºC) | 1 | 0.37 | 0.087 | 1.91 | **0.010** |
| Flower density | 1 | 0.18 | 0.043 | 0.94 | 0.559 |
| Flower species richness | 1 | 0.27 | 0.064 | 1.39 | 0.119 |
| Mean Annual Precipitation (mm) | 1 | 0.18 | 0.043 | 0.94 | 0.548 |
| Residual | 12 | 2.33 | 0.55 |  |  |
