# Supplementary material for: Changes in the structure and composition of the ‘Mexical’ scrubland bee community along an elevational gradient
Source: PLoS One. 2021 Jul 1;16(7):e0254072. doi: 10.1371/journal.pone.0254072 (PMC8248643; doi:10.1371/journal.pone.0254072)
Supplement: S5 Appendix — (DOCX) [file pone.0254072.s005.docx]

**S5 Appendix. Results considering elevation as a categorical variable (instead of continuous).**

Linear Model (GLS) Analyses

**Fig A. Effects of elevation.** Variation of different response variables vs elevation: (A) bee species richness (number of bee species); (B) bee abundance (number of bee individuals); (C) Mean Annual Temperature (MAT, ºC); (D) Mean Annual Precipitation (MAP, mm); (E) flower species richness (number of flower species); (F) flower density (number of flowers/m2); along three defined categories of elevation (‘low’, ‘mid’, ‘high’). All values are ‘mean ± standar error (se)’. NS: Not significant.


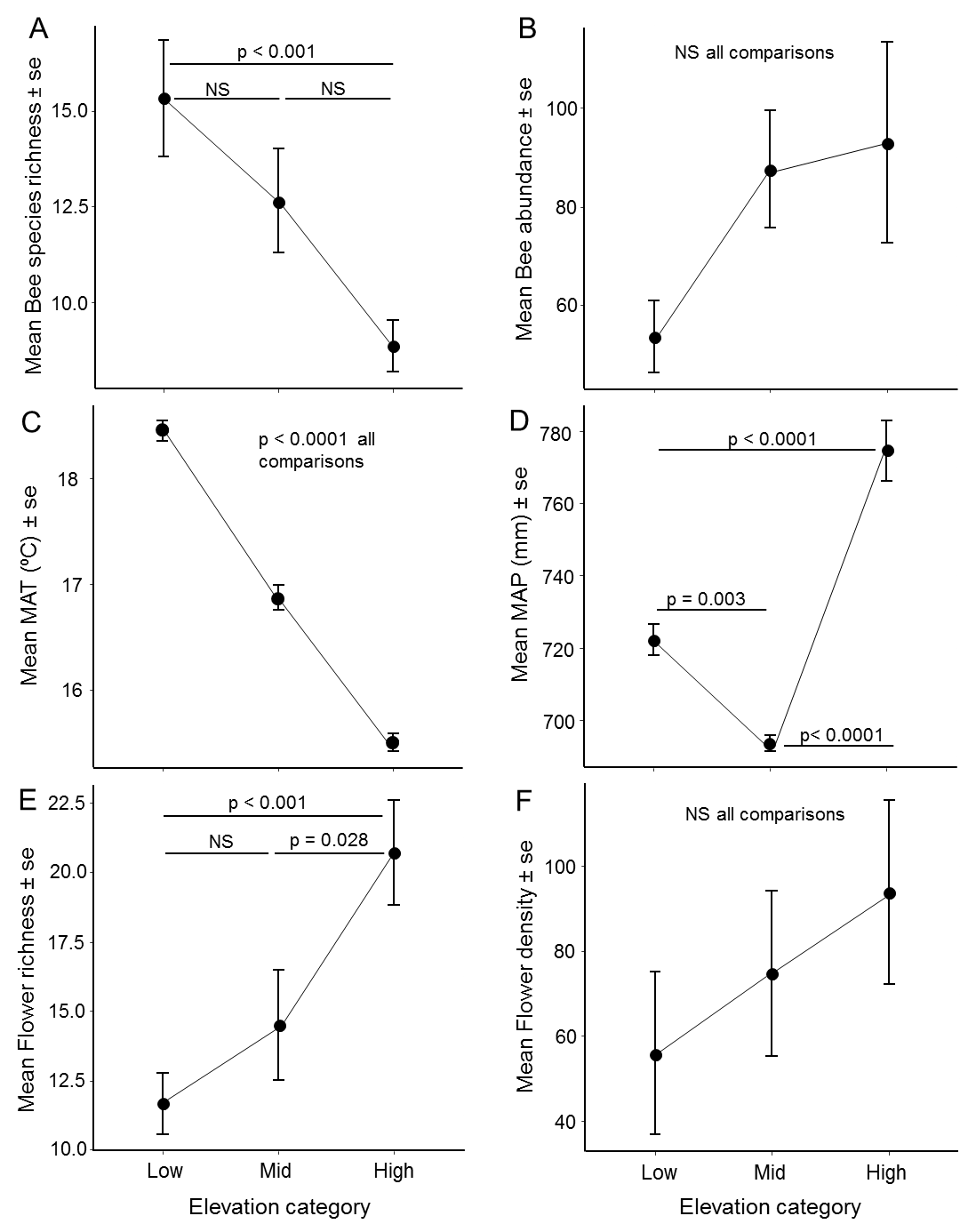


**Table A. Best GLS models for different response variables vs elevation.**

| **GLS model parameters** | | | | **Tuckey Contrasts** | | | | | |
| --- | --- | --- | --- | --- | --- | --- | --- | --- | --- |
|  |  |  |  | low-high | | mid-high | | mid-low | |
| GLS Model | F | p-value | pseudo-R^2^ | z-value | P(>\|z\|) | z-value | P(>\|z\|) | z-value | P(>\|z\|) |
| Bee species richness ~ Elevation | 7.71 | **0.0045** | 0.49 | 3.88 | **<0.001** | 2.29 | 0.06 | -1.54 | 0.27 |
| Bee abundance ~ Elevation | 1.95 | 0.18 | 0.19 | -1.85 | 0.15 | -0.26 | 0.96 | 1.54 | 0.27 |
| MAT ~ Elevation | 217.74 | **<0.0001** | 0.96 | 20.86 | **<0.0001** | 9.69 | **<0.0001** | -10.77 | **<0.0001** |
| MAP ~ Elevation | 47.86 | **<0.0001** | 0.86 | -6.19 | **<0.0001** | -9.58 | **<0.0001** | -3.27 | **0.0031** |
| Flower species richness ~ Elevation | 7.35 | **0.0054** | 0.48 | -3.72 | **<0.001** | -2.55 | **0.028** | 1.22 | 0.50 |
| Flower density ~ Elevation | 0.88 | 0.43 | 0.09 | -1.32 | 0.38 | -0.67 | 0.78 | 0.63 | 0.80 |

Best GLS models for different response variables vs elevation (after controlling for spatial autocorrelation). Parameters of models and Tuckey contrast among the three categories of variable elevation (‘low’, ‘mid’, ‘high’) for each model are presented. (These results are plotted in Fig 2). Significant values are marked in bold. Abbreviations: MAT: Mean Annual Temperature (ºC); MAP: Mean Annual Precipitation (mm).

Multivariate Analyses (Community Composition vs Elevation and different variables)

We present only those analyses that involve variable Elevation, considering it as a categorical variable. These analyses are PERMANOVA (considering only Elevation as explanatory variable) and dbRDA analyses (considering Elevation + geographic distance variables (pcnm1 and pcnm6) as explanatory variables). In all cases community composition is response variable, and quantitative matrix and Bray-Curtis dissimilarity index is applied.

**Table B. Community composition vs Elevation (as a categorical variable), and geographical distance, for complete quantitative matrix data.**

| **PERMANOVA** |  |  |  |  |  |
| --- | --- | --- | --- | --- | --- |
| variable | Df | Sum of Squares | R^2^ | F | P(>F) |
| Elevation | 2 | 0.93 | 0.31 | 3.53 | **0.001** |
| Residual | 16 | 2.12 | 0.69 |  |  |
| **dbRDA** (controlling for geographic distance) | | |  |  |  |
| variable | Df | Sum of Squares | R^2^ | F | P(>F) |
| pcnm1 | 1 | 0.24 | 0.08 | 2.13 | 0.064 |
| pcnm6 | 1 | 0.28 | 0.09 | 2.48 | **0.038** |
| Elevation | 2 | 0.44 | 0.14 | 1.93 | **0.032** |
| Residual | 14 | 1.59 | 0.52 |  |  |

**Table C. Community composition vs Elevation (as a categorical variable), and geographical distance, for quantitative matrix excluding singletons.**

| **PERMANOVA** |  |  |  |  |  |
| --- | --- | --- | --- | --- | --- |
| variable | Df | Sum of Sqs. | R^2^ | F | P(>F) |
| Elevation | 2 | 0.93 | 0.31 | 3.65 | **0.002** |
| Residual | 16 | 2.05 | 0.68 |  |  |
| **dbRDA** (controlling for geographic distance) | | |  |  |  |
| variable | Df | Sum of Sqs. | R^2^ | F | P(>F) |
| pcnm1 | 1 | 0.24 | 0.08 | 2.19 | 0.06 |
| pcnm6 | 1 | 0.28 | 0.09 | 2.57 | **0.028** |
| Elevation | 2 | 0.43 | 0.14 | 1.98 | **0.035** |
| Residual | 14 | 1.53 | 0.51 |  |  |

**Table D. Community composition vs Elevation (as a categorical variable), and geographical distance, for quantitative matrix excluding singletons and**

| **PERMANOVA** |  |  |  |  |  |
| --- | --- | --- | --- | --- | --- |
| variable | Df | Sum of Sqs. | R^2^ | F | P(>F) |
| Altitude | 2 | 0.92 | 0.32 | 3.69 | **0.001** |
| Residual | 16 | 1.99 | 0.68 |  |  |
| **dbRDA** (controlling for geographic distance) | | |  |  |  |
| variable | Df | Sum of Sqs. | R^2^ | F | P(>F) |
| pcnm1 | 1 | 0.23 | 0.079 | 2.18 | **0.047** |
| pcnm6 | 1 | 0.29 | 0.098 | 2.71 | **0.030** |
| Elevation | 2 | 0.42 | 0.143 | 1.98 | **0.027** |
| Residual | 14 | 1.48 | 0.507 |  |  |

**Table E. Community composition vs Elevation (as a categorical variable), and geographical distance, for qualitative (binary) matrix.**

| **PERMANOVA** |  |  |  |  |  |
| --- | --- | --- | --- | --- | --- |
| variable | Df | Sum of Sqs. | R^2^ | F | P(>F) |
| Altitude | 2 | 0.97 | 0.23 | 2.37 | **0.001** |
| Residual | 16 | 3.28 | 0.77 |  |  |
| **dbRDA** (controlling for geographic distance) | | |  |  |  |
| variable | Df | Sum of Sqs. | R^2^ | F | P(>F) |
| pcnm1 | 1 | 0.16 | 0.037 | 0.78 | 0.75 |
| pcnm5 | 1 | 0.27 | 0.063 | 1.33 | 0.14 |
| Elevation | 2 | 0.55 | 0.13 | 1.37 | **0.058** |
| Residual | 14 | 2.83 | 0.66 |  |  |
