# Supplementary material for: Changes in the structure and composition of the ‘Mexical’ scrubland bee community along an elevational gradient
Source: PLoS One. 2021 Jul 1;16(7):e0254072. doi: 10.1371/journal.pone.0254072 (PMC8248643; doi:10.1371/journal.pone.0254072)
Supplement: S6 Appendix — (DOCX) [file pone.0254072.s006.docx]

**S6 Appendix**. Abundance of the three most abundant species in our Mexical community vs elevation, and abundance of bees excluding most abundant species vs elevation. Analyses were conducted considering elevation as continuous and as categorical variable.

**Figure A**. Abundance of the three most abundant especies (*Macrotera sp1* (Figs. A1 and A4), *Lasioglossum (Dialictus) sp1* (Fig. A2 and A5), and *Lasioglossum (Lasioglossum) sp1* (Figs. A3 and A6), considering Elevation as a categorical variable (Figs. A1, A2 and A3; categorical plots represent mean ± standard error), or as a continuous variable (in ‘meters above sea level’: m asl) (Figs, A4, A5, and A6). (Caution: Axes not at the same scale). Significance is shown below each model plot (post-hoc comparisons for categorical plots and p-values for continuous plots; see details of the statistical analyses in Table A on the next page).

Fig.A3

Fig.A2

Fig.A1


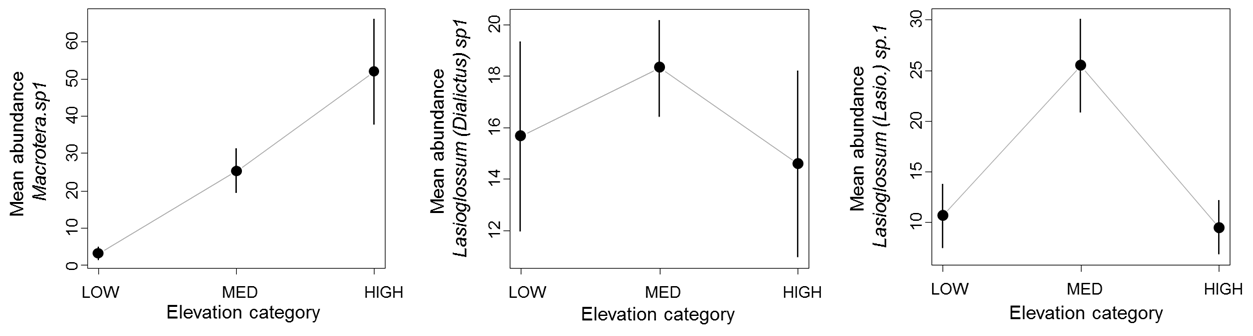


MED-LOW: p=**0.0009** MED-LOW: p=0.84 MED-LOW: p=0.054

HIGH-LOW: p=<**0.0001** HIGH-LOW: p=0.97 HIGH-LOW: p=0.99

HIGH-MED: p=0.8 HIGH-MED: p=0.69 HIGH-MED: p=**0.042**

Fig.A6

Fig.A5

Fig.A4


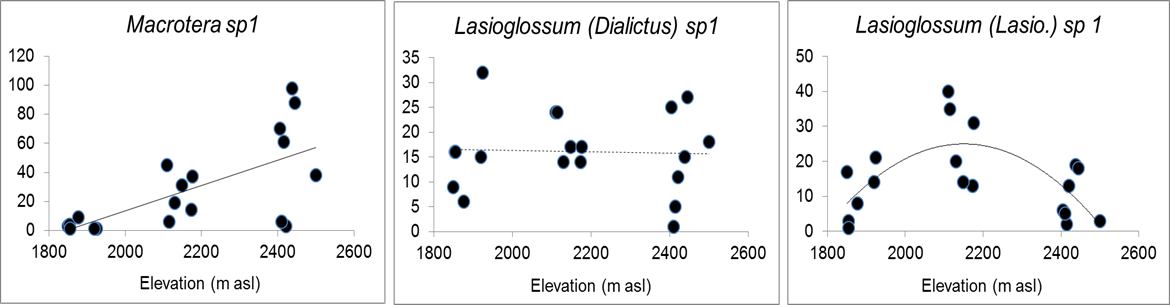


p=**0.001** p=0.868 Elevation: p=0.88

Elevation2: p=**0.0057**

**Table A.** Results for linear models (GLS, controlling for spatial autocorrelation) considering abundance of each of the three most abundant species (*Macrotera sp1*, *Lasioglossum (Dialictus) sp1*, and *Lasioglossum (Lasioglossum) sp1*), abundance without considering the three most abundant species, and abundance without the most abundant species (*Macrotera sp1*), vs Elevation (considering Elevation as a categorical variable (‘cat.’), or as a continuous variable (‘cont.’))

| Models for each individual most abundant species vs Elevation | F | p | pseudoR^2^ |
| --- | --- | --- | --- |
| (Abundance of *Macrotera sp1**^1^) ~ Elevation (cat.) (*2) | 10.79 | **0.001** | 0.57 |
| (Abundance of *Macrotera sp1**^1^) ~ Elevation (cont.) (*2) | 15.33 | **0.001** | 0.47 |
| Abundance of *Lasioglossum(Dialiactus)sp1* ~ Elevation (cat.) | 0.35 | 0.708 | 0.042 |
| Abundance of *Lasioglossum(Dialiactus)sp1* ~ Elevation (cont.) | 0.028 | 0.868 | 0.0017 |
| (Abundance of *Lasioglossum(Lasio.)sp1**^1^) ~ Elevation (cat.) (*2) | 3.68 | **0.048** | 0.32 |
| (Abundance of *Lasioglossum(Lasio.)sp1**^1^) ~ Elevation + Elevation^2^ (cont.) (*2) |  |  | 0.39 |
| Elevation | 0.02 | 0.88 |  |
| Elevation^2^ | 10.14 | **0.0058** |  |
| Models for abundance without considering the three most abundant species (‘3maspp’) vs Elevation |  |  |  |
| (Abundance without 3maspp*^1^) ~ Elevation (cat.) | 0.82 | 0.458 | 0.093 |
| (Abundance without 3maspp*^1^) ~ Elevation (cont.) | 1.66 | 0.213 | 0.089 |
| Models for abundance without considering the most abundant species (*Macrotera sp1*) vs Elevation |  |  |  |
| (Abundance without *Macrotera sp1*) ~ Elevation (cat.) | 1.65 | 0.223 | 0.17 |
| (Abundance without *Macrotera sp1*) ~ Elevation + Elevation^2^ (cont.) |  |  | 0.18 |
| Elevation | 0.8 | 0.38 |  |
| Elevation^2^ | 2.85 | 0.11 |  |

*^1^: log10-transformed

*2: Response variables that showed spatial autocorrelation based in Moran’s I test. In these two auto-correlated response variables, we followed procedure as described in Zuur et al. (2009), to control for spatial auto-correlation. In the case of the remaining response variables (with no spatial autocorrelation), we run a GLS model with no spatial covariance structure and with REML estimation.

**References**

Zuur A, Ieno EN, Walker N, Saveliev AA, Smith GM. Mixed Effects Models and Extensions in Ecology with R. 1st ed. New York: Springer; 2009.
