# Supplementary material for: Changes in the structure and composition of the ‘Mexical’ scrubland bee community along an elevational gradient
Source: PLoS One. 2021 Jul 1;16(7):e0254072. doi: 10.1371/journal.pone.0254072 (PMC8248643; doi:10.1371/journal.pone.0254072)
Supplement: Attachment — Submitted filename: Response to Reviewers.docx [file pone.0254072.s007.docx]

**Response to Reviewers**

***Reviewer #1****:*

*Reviewer #1: I have no further comments. I believe that the authors have addressed my original comments thoroughly.*

**Authors:** Thank you for your comments. We appreciate them.

***Reviewer #2***

*Reviewer #2: Review of the manuscript entitled “Changes in the structure and composition of the ‘Mexical’ scrubland bee community along an altitudinal gradient” for PLOS ONE (PONE-D-20-34943R1).*

*Comments to the author(s)*

*I congratulate the authors for their effort in addressing the comments and suggestions made by me and the other reviewer. In my opinion, the authors solved most of the issues and explained the remaining problems. I have only very minor suggestions to help polishing the manuscript.*

**Authors:** Thank you for your comments. We appreciate them.

*I understood the authors’ explanation for why to maintain the categorical approach in the manuscript. However, in my opinion, the patterns are much clearer to see with the scatter plots and regression curves, specially because we can see the spread of data, which is not possible to visualise with the current plots. The authors could maintain the Figure 4 with the NMDS using the categories just for visualisation purposes and explain that in Methods. Hence, my suggestion is to invert the graphs showed in the manuscript and S8 Appendix, i.e., moving the categorical approach to supplementary material and bringing the continuous approach to the main manuscript. Again, this is just a suggestion and I believe that the decision of presenting the results of continuous or categorical elevation in the main manuscript is up to the authors.*

**Authors:** We have followed the suggestion of the reviewer, and we have included all analyses results and plots considering elevation as continuous variable in the main manuscript, and we have moved all the analyses results and plots considering elevation as categorical variable to supporting information (S5 Appendix in this new version). Further, in the new version, we have reorganized data contained in other Appendixes: we have included all results for multivariate complementary analyses considering elevation as a continuous variable in only one Appendix (information included in S4, S5 and S6 Appendixes in previous version, is now included in the new version in only one Appendix: S4 Appendix). That’s why we have only 6 Appendixes in this new version (instead of 8 Appendixes in the previous version).

Also, as suggested by the reviewer, we have maintained the Figure 4 (NMDS) using variable elevation as three categories for visualization purposes, and we have explained that in ‘Methods’ (see lines 294-297).

*Line 160 – Change to “Hence, our elevation categories…”*

**Authors:** This sentence has been rewritten in the new version on the manuscript (see lines 158-172).

*Line 245 – Change to “For each model, we also obtained an adjusted-pseudo R²…”*

**Authors:** Done. Thank you. (see lines 249-250).

*Line 248 – Change ‘explicative’ to ‘explanatory’.*

**Authors:** Done. Thank you. (see line 262).

*Line 256 – Change to “…response variables i.e., bee species richness and bee abundance, for each one of these two bee…”*

**Authors:** Done. Thank you. (see line 270).

*Line 452 – Change ‘MAT’ to ‘mean annual temperature’.*

**Authors:** Done. Thank you. (see line 483).

*Lines 474 to 483 – I think this paragraph could come after the next one (from lines 484 to 500). In addition, I suggest the authors including one or two sentences in Methods’ section to explain why and how they did these analyses.*

**Authors:** We have relocated the first paragraph (lines 474 to 483 in previous version) after the next one (lines 484 to 500 in previous version), following reviewer’s suggestion (see now lines 523-532 in the new version of the manuscript).

As the reviewer suggests, we also have included a couple of sentences in Methods’ section to explain why and how we did these analyses (see lines 252-261). Details for these analyses and results for these models are shown in the S6 Appendix.
